# O Serogroup-Specific Touchdown-Multiplex Polymerase Chain Reaction for Detection and Identification of *Vibrio cholerae* O1, O139, and Non-O1/Non-O139

**DOI:** 10.1155/2014/295421

**Published:** 2014-12-28

**Authors:** Adisak Bhumiratana, Achiraya Siriphap, Nutsarin Khamsuwan, Jednipit Borthong, Kaknokrat Chonsin, Orasa Sutheinkul

**Affiliations:** ^1^Department of Parasitology and Entomology, Faculty of Public Health, Mahidol University, Bangkok 10400, Thailand; ^2^Department of Microbiology and Parasitology, Faculty of Medical Science, University of Phayao, Phayao 56000, Thailand; ^3^Department of Microbiology, Faculty of Public Health, Mahidol University, Bangkok 10400, Thailand; ^4^Division of Bioinformatics, Research Center for Zoonosis Control, Hokkaido University, Sapporo 001-0020, Japan; ^5^Community Health Program, Faculty of Science and Technology, Suratthani Rajabhat University, Surat Thani 84100, Thailand; ^6^Department of Microbiology, Faculty of Public Health, Thammasat University, Rangsit Center, Pathum Thani 12121, Thailand

## Abstract

A novel, sensitive locus-specific touchdown-multiplex polymerase chain reaction (TMPCR), which is based on two-stage amplification pertaining to multiplex PCR and conditional touchdown strategy, was used in detecting and differentiating *Vibrio cholerae* serogroups. A panel of molecular marker-based TMPCR method generates reproducible profiles of *V. cholerae*-specific (588 bp) amplicons derived from *omp*W gene encoding the outer membrane protein and serogroup-specific amplicons, 364 bp for the O1 and 256 bp for the O139, authentically copied from *rfb* genes responsible for the lipopolysaccharide biosynthesis. The TMPCR amplification efficiency yields either equally or unequally detectable duplex DNA bands of the O1 (588 and 364 bp) and O139 (588 and 256 bp) or a DNA fragment of non-O1/non-O139 (588 bp) while providing no false positive identifications using the genomic DNA templates of the other vibrios and Enterobacteriaceae. The reciprocal analysis of two-template combinations demonstrated that, using *V. cholerae* O1, O139, or equally mixed O1 and O139, the TMPCR had a detection limit of as low as 100 pg of the O1, O139, or non-O1/non-O139 in reactions containing unequally or equally mixed gDNAs. In addition, the O serogroup-specific TMPCR method had 100% agreement with the serotyping method when examined for the serotyped *V. cholerae* reference strains and those recovered from clinical samples. The potential benefit of using this TMPCR tool would augment the serotyping method used in epidemiological surveillance and monitoring of *V. cholerae* serogroups, O1, O139, and non-O1/non-O139 present in clinical and environmental samples.

## 1. Introduction

*Vibrio cholerae* is a water-borne agent causing cholera and is autochthonous to aquatic habitats in coastal and estuarine ecosystems to which symbiosis with zooplanktons for its survival and multiplication is related [1–5].* Vibrio* spp., including* V. cholerae* serogroups [6] and* Vibrio parahaemolyticus* [7, 8], normally grow in the natural environments and can enter into viable-but-nonculturable (VBNC) state. The significant roles of the toxigenic O1 and O139 serogroups of* V. cholerae*, as well as the non-O1/non-O139 serogroups, have been studied in several ways. For instance, the VBNC* V. cholerae* O1 in microcosms or from aquatic environments are converted to culturable state through animal passage [9]. The VBNC O139 and non-O1/non-O139 can also resuscitate when cocultured with several animal cell lines [10, 11]. More recently, the classical biotype of* V. cholerae* O1 retains viability but loses culturability when cocultured with the El Tor biotype [12]. This might suggest that the emergence of the El Tor biotype of* V. cholerae* O1 relates to displacement of the existing classical biotype as the predominant cause of epidemic cholera. Similar to the toxigenic O1 and O139 serogroups that possess virulence-association genes [13–18], the other non-O1 and non-O139 serogroups recovered from aquatic environments or clinical specimens have also been epidemiologically linked with the pathogenic and epidemic potential [18–20]. Thus, public health surveillance and monitoring of cholera cases require the systems used in the national surveillance for notifiable diseases, public health laboratory, and environmental surveillance [21–24]. Molecular detection techniques such as molecular marker-based polymerase chain reaction (PCR) methods developed for probing these eccentric* V. cholerae* microorganisms have been so far proven useful in diagnosis and surveillance for the outbreaks or epidemic investigations worldwide.

In the Gulf of Bengal, South and Southeast Asia, it has been suggested that such outbreaks of cholera pertaining to cross-contamination and spread of* V. cholerae* after the passage(s) from susceptible persons to aquatic environments are likely to be epidemiologically linked with the interconnections of sanitation with poorly chlorinated waters, contamination in seafood/food commodities and seawaters, and direct fecal-oral contact among food handlers or seafood preprocessing plant workers [2, 14–16, 21, 22, 24]. Standard culture methods used in routine diagnosis and surveillance for* V. cholerae* by public health reference laboratories depend on handling the samples of which* V. cholerae* culture is recovered. Regarding this,* V. cholerae* cultures under study investigation were recovered from* V. cholerae*-contracted or suspected patients with acute or severe diarrhea whose stools or rectal swabs had been collectively obtained by clinical laboratory settings during the outbreaks over two past decades between 1994 and 2010 in Central Thailand.

We proposed that the genomic DNA archived from the preserved stock cultures could serve as the template when examined for the O1, O139, and non-O1/non-O139 serogroups by PCR methods as this can also permit a reciprocal detection of the O1 and O139 serogroups from clinical specimens associated with the past outbreaks. In this study, we have successfully developed a novel sensitive, specific, semiquantitative touchdown-multiplex PCR (TMPCR) for detection and differentiation of* V. cholerae* O1, O139, and non-O1/non-O139 serogroups. TMPCR employs the useful molecular markers originally derived from the DNA locus involved in* de novo* O-antigen biosynthesis of* V. cholerae* [25–28] and the outer membrane protein (*omp*W) encoding gene specific for* V. cholerae* [29, 30]. To achieve the goal of the study, we analyzed the performance efficiency (specificity and sensitivity) of this O serogroup-specific TMPCR in detecting and differentiating the O1, O139, and non-O1/non-O139 genomes, which were empirically determined using reference strains of* V. cholerae* and unrelated* Vibrio* strains. We then explored its usefulness in differentiating the O serogroups present in those* V. cholerae* stock cultures originally isolated from clinical specimens. Additionally, the advancement of TMPCR that tested* V. cholerae* present in environmental samples was also discussed.

## 2. Materials and Methods

### 2.1. Bacterial Cultures and Laboratory Classification

Sixty-nine* V. cholerae *strains that served as target gDNA templates included reference and wild-type strains of the O1 (including El Tor/Classical Ogawa and El Tor/Classical Inaba biotypes), O139, and non-O1/non-O139. The 31 other bacterial strains used as nonspecific gDNA templates included 8* Vibrio* spp., 16 Gram-negative bacteria, and 7 Gram-positive bacteria. For the batch propagation of* V. cholerae*, the 0.5 mL overnight culture of the O1 569B or O139 MO45 strains was inoculated into a 100 mL Luria-Bertani (LB) broth supplemented with 1% sodium chloride (NaCl) solution and then incubated at 37°C for 3 h with vigorous shaking. The cell culture with an optical density of 0.8 was used for drop plate method and, subsequently, enumerated by using plate count (PC) agar (Difco, Michigan, USA, or Eiken, Japan). The PC agar was also supplemented with 1% NaCl. The non-O1/non-O139 strains including O22 and O155 were cultured as before.

Subcultures of* V. cholerae* and other* Vibrio* spp. were grown on selective thiosulfate citrate bile salt sucrose (TCBS) agar (Merck, Darmstadt, Germany) and, subsequently, they were biochemically and serologically characterized according to the methods described elsewhere [31, 32]. Briefly, biochemical tests included triple sugar iron (TSI), motility indole-lysine (MIL), oxidase, urea, MR, Voges-Proskauer (VP), citrate, lysine, ornithine, arginine, lactose, sucrose, mannose, arabinose, mannitol, glucose/gas, inositol, aesculin, and salt tolerance of 0%, 3%, 6%, 8%, and 10% NaCl solution. All reagents were purchased from Difco Laboratory (Difco, Michigan, USA). The serogroups O1, O139, and non-O1/non-O139 were tested on the basis of agglutination reaction using commercially available O serogroup-specific antisera (antiserum VcO1/O139 Polyvalent SAP, S & A Reagents Lab, Bangkok, Thailand). The non-O1/non-O139 was characterized into two nonagglutinable (NAG) groups (NAG I and II) according to Heiberg's reaction. In addition, PC agar was used for viable count and enumeration of all the bacterial strains tested in this study.

### 2.2. *V. cholerae *Cultures from Clinical Specimens and Serotyping

A total of 148 stock cultures of* V. cholerae* that corresponded to 108 stools and 40 rectal swabs were collectively obtained from* V. cholerae*-contracted or suspected patients with acute or severe diarrhea during the outbreaks in Central Thailand between 1994 and 2010. By using anonymous system, the cultures initially isolated by clinical laboratory settings were performed on isolation source and time of collection and the corresponding TCBS stock cultures were prepared. All were subsequently subcultured and kept in semisolid agar medium containing 1% NaCl at the Department of Microbiology, Faculty of Public Health, Mahidol University, Thailand. For serotyping the O1, O139, and non-O1/non-O139, the agglutination of the 148 subcultured* V. cholerae* isolates in individuals was blindly performed as before. In addition, only the* V. cholerae *O1 serogroup whose subcultures agglutinated with polyvalent O1 antiserum was characterized using monovalent Ogawa or Inaba antiserum. For biotyping, the phenotypic testing of the strains was performed using hemolysis of sheep blood, agglutination of chicken erythrocytes, Voges-Proskauer, and sensitivity to polymyxin B. Meanwhile, the genomic DNAs of the same* V. cholerae* isolates were prepared according to the rapid boiling method described below or using QIAamp DNA minikit (Qiagen, Hilden, Germany). The stock solution of purified gDNA extracts was initially quantified and then stored according to the method described below.

### 2.3. Genomic DNA Extraction

A modified miniprep gDNA extraction method was used for both target and nontarget gDNAs as follows. As mentioned above, the bacterial culture (1.5 mL) that was grown overnight at 37°C with vigorous shaking was spun down at 12,000 rpm for 1 min. The cell pellet was suspended in 582 *μ*L of TE buffer (10 mM Tris-HCl, 1 mM EDTA), pH 8.0. The suspension was lysed with an addition of 15 *μ*L 20% sodium dodecyl sulfate solution and 3 *μ*L 20 mg/mL proteinase K, mixed thoroughly, and then left at 37°C for 1 h. A 10 *μ*L of 10 mg/mL RNase A was added, mixed thoroughly, and incubated at 37°C for 30 min. The 100 *μ*L 5 M sodium chloride (NaCl) solution and 80 *μ*L 10% hexadecyl trimethyl ammonium bromide (CTAB) in 0.7 M NaCl solution were added, mixed thoroughly, and incubated at 65°C for 10 min. Then, a 0.4x volume of chloroform/isoamyl alcohol (24 : 1) was added, mixed gently for 5 min, and centrifuged at 12,000 rpm for 5 min. The aqueous phase containing gDNA was twice treated with an equal volume of phenol-chloroform and followed by centrifugation as before. An equal volume of isopropanol was added to recovered DNA solution, mixed thoroughly, and DNA pellet was centrifuged as before. After dehydration of an extreme volume of 70% ice-cold ethanol and further centrifugation, it was dissolved in TE buffer pH 7.6.

In addition, using rapid boiling method, the same overnight culture was spun down at 12,000 rpm for 1 min. The cell pellet was suspended well in 500 *μ*L of TE buffer pH 8.0 and incubated at 56°C for 30 min. Immediately after being boiled at 100°C for 10 min, the suspension was stood on ice for 10 min and then centrifuged at 12,000 rpm for 3 min to pellet cell debris; the clear gDNA supernate was obtained. Finally, the purified gDNA extracts as well as crude extracts (boiled lysate) were qualitatively and quantitatively analyzed by spectrophotometer at a wavelength ratio of 260/280 nm or by agarose gel electrophoresis. The aliquots of varying concentration of 30 to 50 *μ*g/mL (a 260/280 OD greater than 1.7) used throughout the study were kept at −20°C until use.

### 2.4. Primer Design

The homologous primer sets specific for* V. cholerae *serogroups (Table 1) were originally derived from target DNA sequences of* V. cholerae *O1, a 22 kb* rfb* DNA locus [25], and of* V. cholerae *O139, a 46 kb* rfb* DNA locus [28]. These* rfb *gene homologs are involved in lipopolysaccharide (LPS) biosynthesis for the O1 and O139 antigens. The* rfb*V gene responsible for the* rhs *element involved in LPS biosynthesis was designed for* V. cholerae *O1-specific primers to generate 364 bp amplicon. The* wbf*R gene encoding asparagine synthetase was designated as* V. cholerae *O139-specific primers (256 bp amplicon). The* omp*W gene encoding the outer membrane protein was used for* V. cholerae *species-specific or universal primers that amplify 588 bp amplicons [29, 30]. Analysis of their unanimous homology at DNA and protein levels was performed on the online available BLAST Programs (http://www.ncbi.nlm.nih.gov/blast/), BLASTN, BLASTx, and BLASTP, for sequence similarity algorithms. The multiple sequence alignments were analyzed for consensus sequences using the ClustalW Program (http://www.ebi.ac.uk/clustalW/). The aliquots of these synthesized multiplex primer sets at a stock concentration of 100 *μ*M in TE buffer pH 8.0 used throughout the study were stored at −80°C until use.

### 2.5. Touchdown-Multiplex PCR Amplification

Initially, two-template combinations of the* V. cholerae *O1 and O139 were tested under the simultaneous locus-specific amplification conditions [33, 34] using a 96-well MyCycler Thermal Cycler (BIORAD, USA) or PCR gradient thermal cycler (ThermoHybraid Px2, Ashford, UK), in which two combined factors such as concentrations of primer sets and gDNA templates were empirically determined in a 25 *μ*L PCR mixture consisting of 1x PCR buffer (50 mM KCl, 1.5 mM MgCl_2_, and 10 mM Tris-HCl pH 9.0), 200 *μ*M each dNTP (dATP, dTTP, dCTP, and dGTP), 1 U of AmpliTaq (Perkin Elmer, USA), or* Taq *DNA polymerase (Promega, USA). The amplification conditions were performed on predenaturation at 95°C for 5 min and followed by 30 cycles of denaturation at 94°C for 1 min and extension at 72°C for 1 min, except that primer annealing temperature decrements (70 → 50°C) were used each for 1 min. The last extension was done at 72°C for 10 min. First, using a constant concentration of 1 *μ*M each primer set, reactions employed mixed O1 569B and O139 MO45 gDNAs (ng) at equal ratios of 10 : 10, 1 : 1, and 0.1 : 0.1 or at unequal ratios of 1 : 10 and 0.1 : 10 and* vice versa*. Second, using mixed gDNAs (10 ng each), the primer sets (universal : O1-specific : O139-specific) at concentration ratios (*μ*M) of 1 : 1 : 1, 1 : 0.5 : 0.5, 1.0 : 0.2 : 0.2, 0.5 : 1.0 : 1.0, 0.5 : 0.5 : 1.0, 0.2 : 0.2 : 1.0, and 0.1 : 0.1 : 1.0 were analyzed. Under these circumstances, the primer binding specificity in amplifying target loci depended upon annealing temperature algorithms to which primer-template combinations were restricted. These algorithms permitted the development of thermocycling TM-PCR protocol as described below.

Finally, a 25 *μ*L optimized TMPCR mixture was similar except that* V. cholerae *O1 and O139 gDNA templates, 10 ng each, and three multiplex primer sets (0.4, 0.4, and 1.0 *μ*M each, resp.) were used instead. The optimized amplification condition was performed on one cycle of predenaturation at 94°C for 5 min, followed by the 5 cycles with successive annealing temperature decrements that one °C changed from 57°C to 53°C in every cycle versus time increments (30 sec in the first three cycles and 35 sec in the last two cycles). The reaction was denatured at 94°C for 1 min, followed by annealing at this temperature algorithm and polymerization at 72°C for 1 min. Subsequently, the 20 cycles of denaturation at 94°C for 1 min, annealing at 52°C for 40 sec, and elongation at 72°C for 1 min were employed. The last primer extension step was done at 72°C for 10 min. In similar fashion, the purified* V. cholerae *O22 or O155 gDNA was used instead of the O139. Either* Escherichia coli *gDNA template as negative control (NC) or nuclease-free water, instead of gDNA template, as internal control (IC) was used in all the experiments.

### 2.6. Semiquantitative TMPCR

The reciprocal detection of* V. cholerae *O1 versus O139 was aimed at analyzing the amplification efficiency of the O serogroup-specific TMPCR under carefully controlled experiments that utilized gDNA template ratios whether unequally or equally. That is, the detectable level of O1 gDNA could be amplified in reactions containing high amount of other related O139 gDNAs, and* vice versa*.

The unequally reciprocal detection of* V. cholerae *O1 versus O139 (Table 2) was done as follows. In experiment A, the reactions contained the amount of the 100 ng O1 gDNA versus the amount of 10-fold serially diluted O139 gDNA (100 ng to 1 pg), or the amount ratios of the O1 : O139 varied from onefold to 10^5^-fold, and* vice versa*. In experiment B, the reactions contained the amount ratios of the O1 : O139 gDNAs as 100 ng to 1 pg, 10 ng to 10 pg, and 1 ng to 100 pg or the amount of the O1 as 10^5^-, 10^3^-, and 10-fold greater than the O139, and* vice versa*. Also, the amount of the 100 pg O139 gDNA was tested against the varying amounts of the O1 gDNA: 100 ng, 50 ng, 25 ng, 10 ng, 1 ng, and 100 pg.

The equally reciprocal detection of* V. cholerae *O1 versus O139 (Table 3) was done as follows. In experiment C, the equal amounts of the O1 : O139 gDNAs were tested at a serial 10-fold dilution, 100 ng to 1 pg. For the internal controls (experiments D and E), the same amounts of the 10-fold serially diluted gDNA templates, O1 or O139, were used. Also, the purified* V. cholerae *O22 or O155 gDNA template was used instead of O139. All experiments were performed in triplicate.

### 2.7. 16S rDNA Amplification

For the quality control of the TMPCR amplification, the 16s rDNA amplification that served as internal control reaction was performed in triplicate with all the reactions containing target or nontarget DNA template throughout the study. In addition, the primers that are specific for microbial DNA (16S rDNA locus) included forward primer 5′-CGG TGA AAT GCG TAG AGA T-3′ and reverse primer 5′-TTA CTA GCG ATT CCG AGT TC-3′ [35]. A 25 *μ*L optimized PCR mixture contained 1X PCR buffer and 200 *μ*M each dNTP as before except that forward and reverse primers,* Taq* DNA polymerase, and gDNA template were used at concentration of 0.5 *μ*M each, 0.5 U, and 1 *μ*L of undiluted template, respectively. The optimized amplification was performed on predenaturing at 94°C for 5 min and followed by 30 cycles of denaturing at 94°C for 45 sec, annealing at 55°C for 45 sec, and extension at 72°C for 45 sec. Finally, the last extension was at 72°C for 5 min. In this regard, the amplicons with expected size of 663 bp were obtained from all reactions containing microbial gDNA templates.

### 2.8. Analysis of PCR Products

One-fifth of the amplicons mixed with 6x loading dye solution were electrophoresed through the 1.5% to 2.0% agarose gels at constant voltage of 10 V/cm in 1x TBE buffer (89 mM Tris-HCl, 89 mM Borate, 2 mM EDTA) (Ameresco, Ohio, USA). The ethidium bromide- (EtBr-) stained DNA gel was used to analyze the amplicon sizes in base pairs as compared to DNA standard size marker (500 ng of each 100 bp ladder) (Phamacia Biotech, USA). The intensity of EtBr-stained DNA bands was visualized and photographed under an ultraviolet (UV) wavelength using DNA gel documentation (Fotodyne, Hartland, USA).

In addition, analysis of amplification specificity, sensitivity, and fidelity of the O serogroup-specific TMPCR was done as follows. In reactions using target gDNAs, intense EtBr-stained duplex bands were detected, either equally or unequally, for O1 (588 bp and 364 bp) and for O139 (588 bp and 258 bp). An intense 588 bp band was detected for the non-O1 (both NAG I and NAG II). In reactions using nontarget gDNAs or nuclease-free water instead of DNA, neither expected amplicon sizes nor nonspecific DNA bands were observed.

## 3. Results

**Figure 1:**
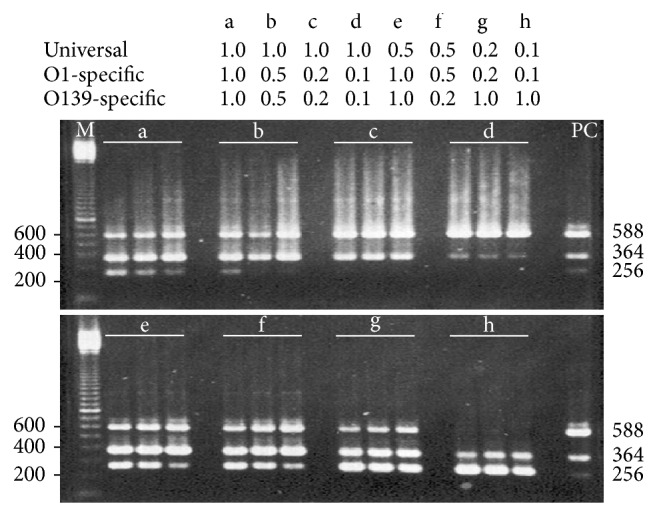
Agarose gel electrophoresis analysis of the PCR algorithm upon annealing temperatures and primer set concentrations that had effects on yields of three specific amplicons. In a 25* μ*L PCR assay using 10 ng each of purified* V. cholerae* O1 and O139 gDNAs, amplification was performed in triplicate with varying concentration of three primer sets (*μ*M) (a to h) and annealing temperature increments (°C) at 54.2, 55.5, and 56.9. The 100 bp molecular marker (lane M) and serogroup-specific PCR amplicons as positive control (lane PC) were used for base pair size comparisons.

Primer-template specificity binding was empirically determined in reactions of which optimized PCR parameters were analyzed upon the amplification efficiency of three multiplex primer sets that specifically bind to* V. cholerae* gDNAs of the O1, O139, or O22/O155. We initially optimized the separate reactions containing the gDNA content of* V. cholerae* O1 569B or O139 MO45 alone, or equally mixed. Most reliable amplification conditions were reproducibly performed on the reactions using a 10 ng each of* V. cholerae* O1 569B or O139 MO45 per reaction assay, or equally mixed. The resulting algorithm upon annealing temperature above 55°C was likely to decrease amplification yields of the specific amplicons with expected sizes (data not shown). All reactions containing O22 or O155 gDNA putatively yielded a 588 bp DNA band (data not shown). Optimum annealing temperatures ranged from 53°C to 57°C. It was clear that PCR factors underlying the annealing temperatures and primer set concentrations were more likely to influence the amplification efficiency whether the O1, O139, or equally mixed gDNAs were used. As a result, optimized multiplex PCR amplification depended on annealing temperature at 55°C and primer set concentrations of 0.4 (universal), 0.4 (O1-specific), and 1.0 (O139-specific) *μ*M (Figure 1).

**Figure 2:**
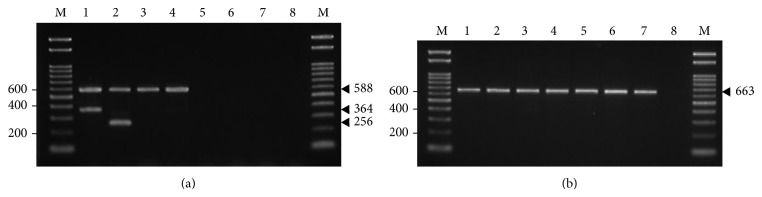
Agarose gel electrophoresis of the amplification specificity of optimized TMPCR (a) and 16S rDNA PCR (b), using representative target and nontarget gDNAs. In lanes 1 to 8, amplification reactions contained* V. cholerae *O1,* V. cholerae *O139,* V. cholerae *NAG I,* V. cholerae *NAG II,* Vibrio parahaemolyticus*,* Vibrio vulnificus*,* Escherichia coli*, and nuclease-free water, respectively. The expected sizes of specific amplicons (base pairs) were compared to the 100 bp standard ladder marker (lane M).

We further employed the stringent protocol developed for TMPCR because the multiplex PCR provided a ladder-like problem of amplification background of most reactions containing nontarget gDNAs. Similarly, the multiplex PCR yielded spurious products even in some reactions containing* V. cholerae* O1, O139, or non-O1/non-O139 gDNAs prepared by CTAB/phenol-chloroform extraction method or by rapid boiling method (data not shown). As a result of optimized O serogroup-specific TMPCR with loose touchdown strategy, thermal cycling condition employed annealing temperature decrements at an interval of 57°C to 53°C versus time increments to minimize nonlagging products during initial geometric amplification cycles. As compared to the 16S rDNA amplification, this proven TMPCR had 100% specificity or gave putatively positive amplifications in reactions containing* V. cholerae* genomes but not other nontarget DNAs (Table 4). Of the 21* V. cholerae* O1 serving as target DNAs tested by the TMPCR, it was clear that consistently positive amplifications with EtBr-intense duplex DNA bands (588 and 364 bp) were observed in reactions containing reference strains (8), Eltor Inaba (5), Eltor Ogawa (4), Classical Inaba (2), and Eltor Ogawa (2). Similarly, other 16* V. cholerae* O139 strains yielded the amplified duplex DNA fragments (588 and 256 bp), while both NAG I and NAG II gave only a single 588 bp DNA band as expected. Only the representative target and nontarget gDNAs amplified by O serogroup-specific TMPCR, as compared to that amplified by 16S rDNA PCR, are shown in Figure 2. In addition, there were no differences of specificity and sensitivity of TMPCR when using* V. cholerae* O1, O139, and non-O1/non-O139 gDNA templates whether purified or boiled.

**Figure 3:**
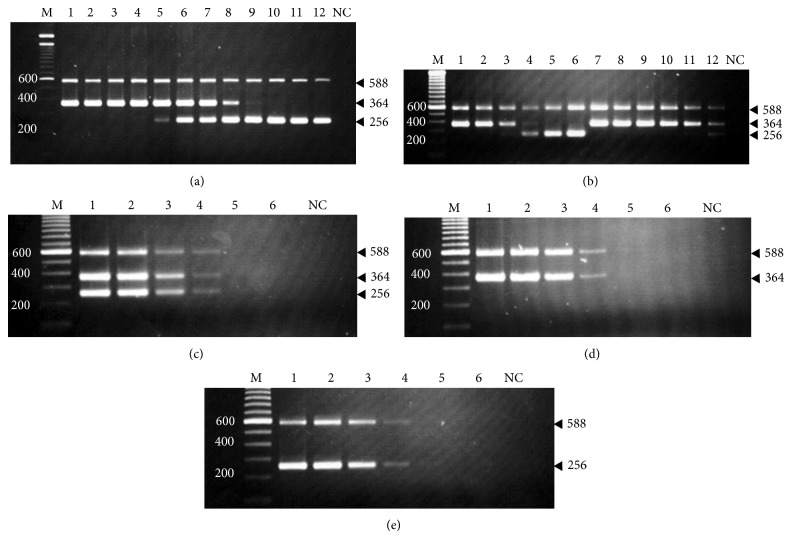
Agarose gel electrophoresis analysis of the amplification efficiency of semiquantitative TMPCR. A reciprocal amplification algorithm of detectable levels of the O1 to O139 gDNAs (experiments (a) to (e)) was described in the text, while* E. coli *gDNA template serving as negative control (NC) was included. (a) Lanes 1 to 6, the 100 ng O1 gDNA versus a 10-fold serially diluted O139 gDNA, 1 pg to 100 ng, and* vice versa* (lanes 7 to 12). (b) Lanes 1 to 3, unequal amount ratios of the O1 : O139, 100 ng to 1 pg, 10 ng to 10 pg, and 1 ng to 100 pg, and* vice versa* (lanes 4 to 6). Lanes 7 to 12, the 100 pg O139 gDNA versus the O1 gDNA contents of varying 100 ng, 50 ng, 25 ng, 10 ng, and 1 ng to 100 pg. (c) Lanes 1 to 6, the equal amount ratios of 10-fold serially diluted O1 to O139 gDNAs, 100 ng to 1 pg. (d) Lanes 1 to 6, 10-fold serially diluted O1 gDNA contents, 100 ng to 1 pg. (e) Lanes 1 to 6, 10-fold serially diluted O139 gDNA contents, 100 ng to 1 pg. A 100 bp DNA ladder marker (lane M) was used in comparison of the amplicons with expected size (bp).

To analyze the amplification sensitivity of the TMPCR, we determined a reciprocal amplification pertaining to the algorithms that used the amount of ratios of mixed* V. cholerae* gDNAs (O1 : O139), in comparison to those reactions containing each gDNA template serially diluted. The resulting TMPCR experiments in which CTAB/phenol-chloroform extracted* V. cholerae* gDNA whether O1, O139, or O22/O155 was amplified are shown in Figure 3. As for Figure 3(a), the TMPCR could detect O serogroup-specific DNA fragments authentically derived from the O1 or the O139 in reciprocal reactions. The detection limit of as low as 1 ng of the O1 was obtained in the presence of the higher amount of O139 or 100-fold greater than the O1. Meanwhile, the detection limit of as low as 10 ng of the O139 was obtained in the presence of the higher amount of the O1 or 10-fold greater than the O139.  Figure 3(b) shows that its detection limit was at 100 pg of the O1 or the O139 in inverse reactions containing unequal amount ratios of the 100 pg O1 to 1 ng O139, and* vice versa*. Again, the TMPCR was likely to show detection limit of as low as 100 pg each of gDNAs in the reaction containing equal amount ratio of the O1 to O139. It was clear to note that the TMPCR had the detection limit of as low as 100 pg gDNA of the O1 or O139 when examined for the equal amount ratio of the serially diluted O1 and O139 templates (Figure 3(c)). Similarly, it also had the same detection limit of as low as 100 pg gDNA of the O1 or O139 when separately copied (Figures 3(d) and 3(e)). The amount of as low as 100 pg O22 or O155 gDNA was also detected (data not shown).

**Figure 4:**
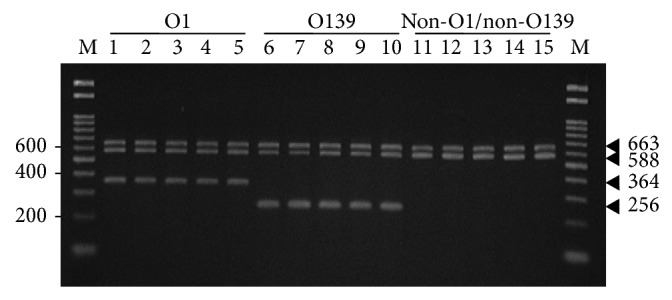
Agarose gel electrophoresis of the O serogroup-specific TMPCR and 16S rDNA PCR profiles using representative* V. cholerae *gDNAs archived from the clinical specimens. PCR products obtained by both TMPCR (5* μ*L) and 16S rDNA PCR (5* μ*L) that were separately performed are confined to the same lane: lanes 1–3, the O1 from stools; lanes 4-5, the O1 from rectal swabs; lanes 6–10, the O139 from stools; and lanes 11–15, the non-O1/non-O139 from stools. In each TMPCR employing the O1, O139, or non-O1/non-O139 gDNA, the reliable reaction represents duplex DNA fragments (588 bp and 364 bp from the O1 or 588 bp and 256 bp from the O139) and a single 588 bp DNA band from the non-O1/non-O139, as compared to 16S rDNA amplicons (663 bp) and 100 bp DNA ladder marker (lane M).

Lastly, we tested the amplification fidelity of O serogroup-specific TMPCR by using target gDNA samples archived from the stock cultures of the 148* V. cholerae* clinical isolates. Overall, the TMPCR exhibited 100% concordance with serotyping method as reference whether* V. cholerae* cultures were obtained from stools or rectal swabs (Table 5). All the 108 cultures from the stools tested gave positive TMPCR results: 65 (60.2%) of the O1 serotype concordant with the O1 serogroup-specific TMPCR (588 and 364 bp), 15 (13.9%) of the O139 serotype concordant with the O139 serogroup-specific TMPCR (588 and 256 bp), and 28 (25.9%) of the non-O1/non-O139 serotype concordant with the O serogroup-specific TMPCR (588 bp). Meanwhile, all the 40 cultures from the rectal swabs were also consistently positive with the tests: 39 (97.5%) accordant with the O1 serotype and the O1 serogroup-specific TMPCR and only one (2.5%) accordant with the non-O1/non-O139 serotype and 588 bp specific TMPCR. Only the representative gDNAs archived from the clinically isolated* V. cholerae* cultures are shown in Figure 4. All samples tested by O serogroup-specific TMPCR were consistently positive with the 16S rDNA PCR.

Additionally, TMPCR provided reliable testing results when examined for* V. cholerae* present in environmental samples such as water samples and seafood, as compared to the culture method/serotyping (Table 6). Particularly when examined for* V. cholerae* present in aquatic environments, TMPCR provided positive result for* V. cholerae* non-O1/non-O139 in a water sample, whereas culture method did not.

## 4. Discussion

The conventional culture technique is fundamental to recover the* V. cholerae* culture from clinical samples of* V. cholerae*-contracted or cholera-like patients. However, the approach relies upon the ability of the* V. cholerae* propagating* in vitro* culture and expressing normally phenotypic characteristics under physiological conditions whether morphologically, biochemically, or serologically. This classical microbiological approach is not just to grow viable microbial cells onto a plate count agar or in an enrichment culture medium which constitutes the entire nutrients essential for growth of the target microbe(s), but also to incubate the propagated cultures under conditions favoring their optimal growth. Such VBNC* V. cholerae* as well as some VBNC vibrios can exist in nature in aquatic habitats such that they are not as yet cultured in the laboratory and hence have not been characterized using routine microbiological methods due to the limits of recovery of resuscitated cultures from environmental samples. However, the* V. cholerae* once recovered from those isolation sources needs to be logically analyzed to determine their property whether phenotypically or genotypically.

In the present study, we provided the proof that all the serotypes of* V. cholerae* both reference strains and clinical isolates were consistently positive with the O serogroup-specific TMPCR method, showing the concordance of serotypes and O serogroup-specific genotypes. In other words, the TMPCR method that employed the primer sets specific for the O1, O139, and non-O1/non-O139 could simultaneously yield amplicons authentically derived from the target DNA sequences only in reactions containing the low amounts of target gDNAs but not the unrelated gDNAs. This implied that the TMPCR had high performance efficiency (specificity and sensitivity) in the detection and identification of the O1, O139, and non-O1/non-O139 serogroups of* V. cholerae* reference strains and clinical isolates. The O serogroup-specific primer sets did not cross-hybridize against nontarget DNA sequences neither in reactions containing target gDNA templates nor in reactions containing unrelated gDNA templates. As for the quantitatively direct determination assessment tool, the TMPCR had the detection limit of as low as 100 pg of* V. cholerae* gDNAs of the O1, O139, or non-O1/non-O139. When mimic conditions of the mixed gDNAs of the O1 and O139 were analyzed, the TMPCR method exhibited high sensitivity. The reaction assays that employed the equally or unequally mixed O1 and O139 gDNAs could compare well with the findings of the reactions that used the single source gDNAs. This might suggest that the O serogroup-specific TMPCR assay could augment the efficiency of the serotyping method when examined for the qualitative and quantitative analyses of the serotyped* V. cholerae* cultures recovered from any isolation sources whether clinically or environmentally. As compared to the culture method, only the TMPCR gave positive result when target DNA of* V. cholerae* non-O1/non-O139 could be detected in a water sample in this study. That is, the TMPCR can provide the proof that* V. cholerae* non-O1/non-O139 present in a water sample is the VBNC state. This promising technique can be used as similar to the molecular markers-based PCR methods that permit the detection and identification of* V. cholerae* O1, O139, and non-O1/non-O139 isolates from clinical patients or aquatic environments [13–24].

Additionally, the TMPCR requires the procedures for the gDNA preparation, target gene amplification, and postanalysis of PCR product. However, compared to that of the serotyping method, the technique becomes easier in terms of process and time required to run a lot of the sampled* V. cholerae* cultures. Similar to that of other touchdown or multiplex PCR methods developed for probing a variety of microorganisms [33, 36, 37], the performance efficiency of the TMPCR tested in this study was achieved by a two-stage amplification incorporating multiplex PCR with conditional touchdown strategies, which were suitable for simultaneous amplification of target DNA sequences with high fidelity and workable rate. This improved multiplex PCR comprises a simultaneous PCR and a specific PCR, and either one or both of the amplification steps are performed with a touchdown strategy, of which loose touchdown strategy is applied with a temperature lower than the optimized annealing temperature, and stringent touchdown strategy is applied with a temperature higher than the optimized annealing temperature. The TMPCR approach to circumvent the problems of multiplex PCR assays has been successfully used for simultaneous amplification of target DNA sequences originally derived from the O1, O139, and non-O1/non-O139 serogroups with the corresponding touchdown-PCR parameters.

## Figures and Tables

**Table 1:** Primer sequences^a^ used in touchdown-multiplex PCR.

Primer set	Sequence (5′ to 3′)	Tm (°C)	Direction	Amplicon size (bp)	Reference
Universal primers^b^					
MVCO1	CAC CAA GAA GGT GAC TTT ATT GTG	68	Forward	588	Nandi et al., 2000 [30]
MVCO2	GAA CTT ATA ACC ACC CGC G	58	Reverse		
O1-specific primers^c^					
MVCO3	GAC TGT CAG CTG GCG GAA	58	Forward	364	This study
MVCO4	GTT GGC GTA TTA CGG TAC	54	Reverse		
O139-specific primers^d^					
MVCO5	GCG TTT ATC GCC GGT CGA C	62	Forward	256	This study
MVCO6	GTA ACT TGG TAC AAT CTC G	54	Reverse		

^a^All were originally derived from the nucleotide sequences (positions) available from the GenBank accession numbers ^b^AE003853 (819612–820199), ^b^CP000626 (404919–405506), ^c^AE003852 (264415–264778), ^c^CP000627 (2789549–2789912), and ^d^U47057 (902–1157); from the EMBL accession numbers ^b^X51948 (221–808), ^c^X59554 (18246–18609), ^c^Y07788 (199–562), and ^d^Y07786 (11878–12133); or from the DDBJ accession number ^d^AB012956 (32652–32907).

**Table 2:** The unequally reciprocal detection of *V*. *cholerae* O1 : O139 by the O serogroup-specific TMPCR.

O1	O139					
	0.001	0.01	0.1	1	10	100
100	10^5^	10^4^	10^3^	10^2^	10^1^	1
10		10^3^				10^1^
1			10^1^			10^2^
0.1				10^1^		10^3^
0.01					10^3^	10^4^
0.001						10^5^

The contents (ng) of purified O1 : O139 gDNAs were used in this assay.

**Table 3:** The equally reciprocal detection of *V*. *cholerae* O1 : O139 by the O serogroup-specific TMPCR.

O1	O139					
	100	10	1	0.1	0.01	0.001
100	10^0^					
10		10^0^				
1			10^0^			
0.1				10^0^		
0.01					10^0^	
0.001						10^0^

The contents (ng) of purified O1 : O139 gDNAs were used in this assay.

**Table 4:** Amplification specificity of TMPCR and 16S rDNA-specific PCR.

Organism	Strains tested (*n* = 100)	Specificity (%)	
		TMPCR^a^	16S rDNA PCR^b^
*V. cholerae *O1	21	100	100
*V. cholerae *O139	16	100	100
*V. cholerae *NAG I^c^	16	100	100
*V. cholerae *NAG II^d^	16	100	100
Other vibrios	8	0	100
Gram-negative bacteria	16	0	100
Gram-positive bacteria	7	0	100

^a^The specificity (%) = strains showing a positive amplification of expected amplicon sizes by TMPCR as having no cross-hybridization with negative controls/a total of strains tested, multiplied by 100.

^b^As for 16S rDNA-specific PCR, no cross-hybridization occurred in reactions containing nuclease-free water or no DNA.

^c^NAG I non-O1/non-O139 growing in semisolid agar in the presence of sucrose and mannose but not arabinose.

^d^NAG II non-O1/non-O139 growing in semisolid agar only supplemented with sucrose.

**Table 5:** Agreement of O serogroup-specific TMPCR for detecting and differentiating gDNAs of the O1, O139, and non-O1/non-O139 serogroups archived from clinical samples (*n* = 148), using serotyping method as reference.

Source	Serogroup^a^	TMPCR		
		*ompW *	*rfbV *	*wbf *
Stool *n* = 108	O1 (*n* = 65)	65	65	0
	O139 (*n* = 15)	15	0	15
	Non-O1/non-O139 (*n* = 28)	28	0	0
	Total	**108**	**65**	**15**

Rectal swab *n* = 40	O1 (*n* = 39)	39	39	0
	O139 (*n* = 0)	0	0	0
	Non-O1/non-O139 (*n* = 1)	1	0	0
	Total	**40**	**39**	**0**

^a^All laboratory settings yielded 100% concordant *V*. *cholerae* cultures whether rectal swabs or stools examined using standard serotyping method as described in the text.

**Table 6:** Advancement of TMPCR for *V*. *cholera* detection using environmental samples.

Source^a^	Sample number	Culture method and serotyping			TMPCR		
		O1	O139	Non-O1/Non-O139	O1	O139	Non-O1/Non-O139
Water samples	50	0	0	0	0	0	1
Seafood	18	5	0	8	5	0	8

^a^All were collectively obtained during the outbreaks. The individual samples enriched in broth were used in *V*. *cholerae* detection and differentiation using the culture method/serotyping and TMPCR.
